# Perioperative management for invasive brain–computer interface implantation under surgical navigation in a patient with traumatic spinal cord injury: a case report

**DOI:** 10.3389/fnhum.2026.1892331

**Published:** 2026-08-25

**Authors:** Shuhui Yang, Qiang Sun, Hong Mo, Wenyao Cui

**Affiliations:** 1 Department of Neurosurgery, West China Hospital, Sichuan University, Chengdu, China; 2 West China School of Nursing, Sichuan University, Chengdu, China; 3 Department of Anesthesiology, West China Hospital, Sichuan University, Chengdu, China

**Keywords:** brain–computer interface, nursing cooperation, perioperative management, spinal cord injury, surgical navigation

## Abstract

**Background:**

The clinical application of invasive brain–computer interface combined with surgical navigation in patients with traumatic spinal cord injury remains limited, and there is a lack of a standardized perioperative nursing cooperation strategy. This paper summarized the intraoperative cooperation and nursing experience from a single case, aim to provide a reference for clinical practice.

**Case summary:**

Preoperatively, the patient presented with a muscle strength of grade 4 in both forearms and upper arms, and grade 0 in both hands. Surgical navigation-assisted invasive brain–computer interface implantation was planned. The operation was successfully completed, and device activation was performed on August 13, 2025. At the 6-month follow-up, the patient’s left-hand brain–computer interface-assisted grasping score was 18 points for the left hand and 4 points for the right hand, with total scores of 11 points for the left hand and 8 points for the right hand, respectively. Improvement in bilateral upper limb motor function was observed. The patient regained voluntary motor function, and the complete paralysis was alleviated. No adverse events occurred during the hospitalization and follow-up period, and the patient and his family members reported a high level of satisfaction.

**Conclusion:**

This paper summarized the perioperative care experience of a patient with traumatic spinal cord injury who underwent an invasive brain–computer interface implantation under surgical navigation. During the perioperative period, refined nursing cooperation was implemented through establishing a multidisciplinary team, conducting comprehensive preoperative preparations, precisely coordinating with surgical navigation, strictly preventing surgical site infections, standardizing postoperative monitoring, and enhancing post-discharge follow-up. The precise perioperative nursing plan is scientifically sound and clinically feasible, providing a practical foundation for the future implementation of perioperative nursing strategies for similar surgical procedures.

## 1 What does this paper contribute to the wider global clinical community?

- Although invasive brain–computer interfaces are rapidly advancing into clinical practice, postoperative infection, immune rejection, electrode displacement, and signal interference remain major safety concerns. This paper establishes a comprehensive nursing protocol that includes preoperative infection prophylaxis, intraoperative navigation nursing collaboration, and postoperative device integrity monitoring, all supported by multidisciplinary teamwork. These evidence-based recommendations address the current lack of standardized nursing guidance for this emerging technology and provide a replicable framework for nursing teams worldwide to mitigate perioperative risks and ensure implantation safety.
- At the 6-month follow-up, the patient’s left-hand brain–computer interface-assisted grasping score was 18 points. This finding is expected to promote the wide application of brain–computer interfaces in patients with spinal cord injuries.

Spinal cord injury (SCI) is a severe injury caused by diverse etiologies that damage the structure and function of the spinal cord, leading to motor, sensory, and autonomic dysfunctions below the injured level (Sterner and Sterner, 2023). SCI can be categorized into traumatic spinal cord injury (TSCI) and non -traumatic spinal cord injury (NTSCI). TSCI refers to spinal cord injury resulting from spinal fracture and/or dislocation caused by direct mechanical force. It is mainly induced by incidents such as traffic accidents, acts of violence, sports-related injuries, or falls (Alizadeh et al., 2019). A study has reported an incidence of TSCI of 49.8 per million population annually (Jiang et al., 2022). Patients with TSCI suffer severe structural and functional impairment. The majority experience long-term disabilities of varying extents, including paralysis, pain, pressure injury, weakness, bladder and bowel incontinence, or respiratory dysfunctions, among other multisystem impairments. Full recovery is difficult to achieve even with long-term rehabilitation training (Patel et al., 2025). TSCI causes substantial physical and psychological distress, impairs patients’ self-management ability, limits mobility and social participation, and severely affects health outcomes and quality of life (AlHuthaifi et al., 2016). Brain–computer interface (BCI) is a human–machine interaction system that establishes a communication pathway between the brain or spinal cord and external devices. It recognizes and outputs the user’s intention by acquiring and decoding central neural activity, controls external devices, and receives feedback to form a closed loop. BCI is mainly applied in the compensation and restoration of human functional impairments, as well as the enhancement of human behavioral capabilities (Young et al., 2021). For patients with limb motor dysfunctions, rehabilitative brain–computer interfaces can provide repetitive stimulation to the brain to reinforce the connections between neurons, thereby achieving partial reconnection or functional repair (Chen et al., 2023). BCI has been listed as a key research priority in the science and technology frontier in China’s *14th Five-Year Plan* and *Outline of Vision 2035*, and is also one of the major project directions of the *Brain Science and Brain-Like Research* initiative under the Science and Technology Innovation 2030 program (People's Republic of China State Council, 2021; Ministry of Science and Technology of the People's Republic of China, 2021). At present, most BCI technologies remain in the experimental research stage, and their clinical application lacks systematic validation.

In July 2025, our hospital performed surgical navigation-assisted invasive brain–computer interface implantation on a patient with TSCI, which was the first case in Western China. After meticulous treatment and nursing care, the patient achieved a satisfactory recovery. The nursing experience is summarized and reported as follows.

## 2 Clinical materials

### 2.1 General information

The patient was a 29-year-old male, with a height of 175 cm and a weight of 70 kg. He was admitted to our department on July 16, 2025, due to sensory and motor dysfunctions of the extremities for more than 2 years. The patient sustained sensory and motor dysfunctions of all four extremities more than 2 years ago, following a sudden jolt while seated in the rear of a car, during which his head struck the vehicle’s roof. On January 19, 2023, he underwent C3–6 posterior laminectomy, laminoplasty, spinal nerve decompression, posterolateral bone fusion with autologous and allogeneic grafts, and pedicle screw fixation from C2 to C7 under general anesthesia. Subsequently, postoperative rehabilitation was prescribed. The patient was admitted to our department for further management. The patient presented with a satisfactory general condition, with normal mental status, appetite, and sleep. Concurrently, the patient exhibited urinary and fecal incontinence, and there were no documented alterations in body weight. Changes. The patient presented with an alert mental state and was cooperative. Bilateral pupils were approximately 2 mm in diameter, exhibiting brisk light reflexes. Visual fields were intact, and tongue protrusion was midline. Muscle strength was graded as 4 in bilateral forearms and upper arms, 0 in bilateral hands and lower extremities. Muscle tone in all extremities was normal. Sensation was absent below the C3 level. Bilateral pathological reflexes were negative, and meningeal signs were negative. Admission diagnosis: 1. Spinal cord injury; 2. Postoperative status of cervical spine surgery.

### 2.2 Treatment course and outcomes

**Figure 1:**
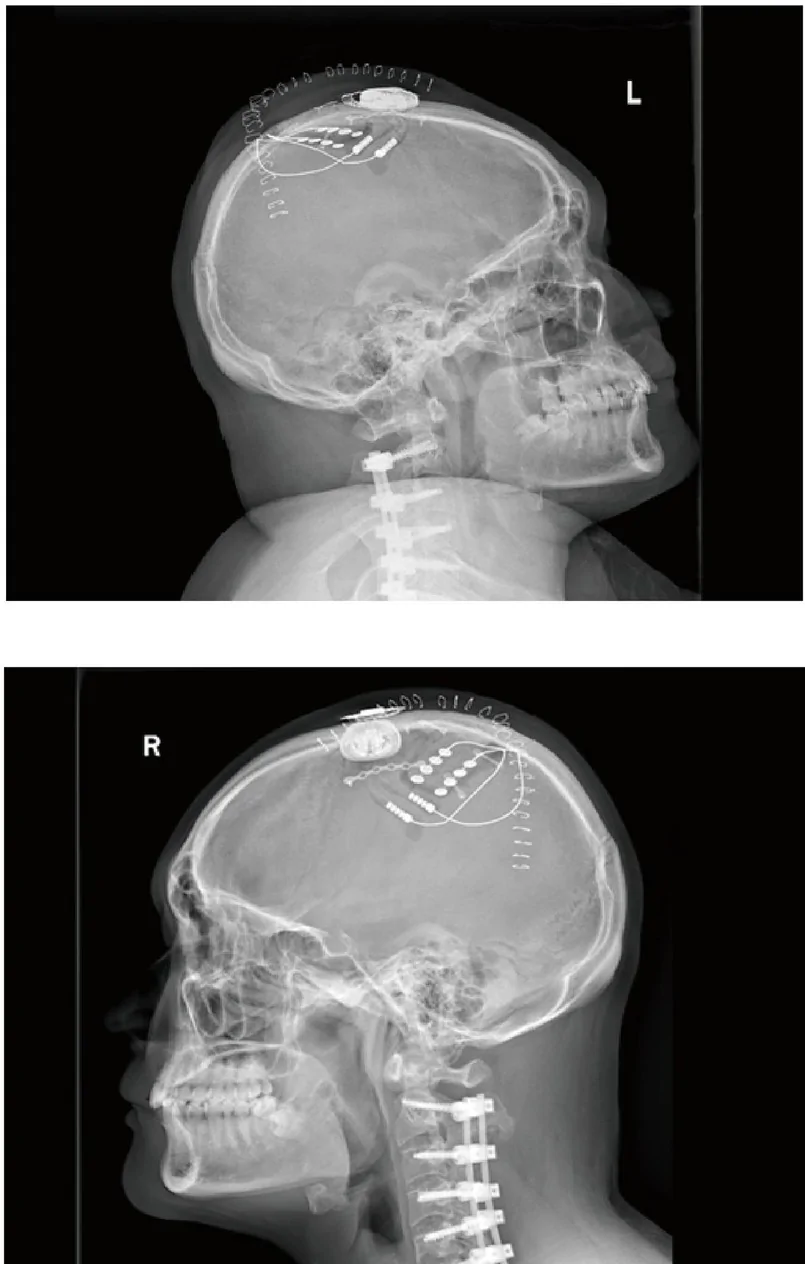
Postoperative imaging data of the patient.

The surgical procedure was performed on July 18, 2025. Following induction of general anesthesia, the patient was positioned supine. Under surgical navigation guidance, the precentral and postcentral gyri were localized, and a frontal curved incision was designed. Routine disinfection and sterile draping were then carried out. The scalp was incised layer by layer, the skin flap was reflected, and subsequently fixed with a spring retractor. A bone flap was created using a high-speed drill and a craniotome, then elevated, after which the dura mater was suspended. With the assistance of surgical navigation and electrophysiological monitoring, the precentral and postcentral gyri were re-localized, and brain–computer interface electrodes were fixed epidurally at the precentral and postcentral gyri, respectively. The bone flap was then repositioned and secured with titanium screws and caps. The electrodes were connected to the main unit of the brain–computer interface, which was subsequently fixed in a subcutaneous pocket. The subcutaneous tissue and skin were sutured in layers. Normal electroencephalographic (EEG) signals were acquired immediately after electrode implantation, confirming the technical success of the procedure. The total operative time was 3 h and 40 min, with an estimated intraoperative blood loss of 50 mL. The patient was uneventfully transferred to the ward after recovery from anesthesia. Postoperative computed tomography (CT) performed on postoperative day 1 confirmed the position of the implant, as shown in Figure 1. The patient was discharged on July 26, 2025, for home-based rehabilitation.

**Figure 2:**
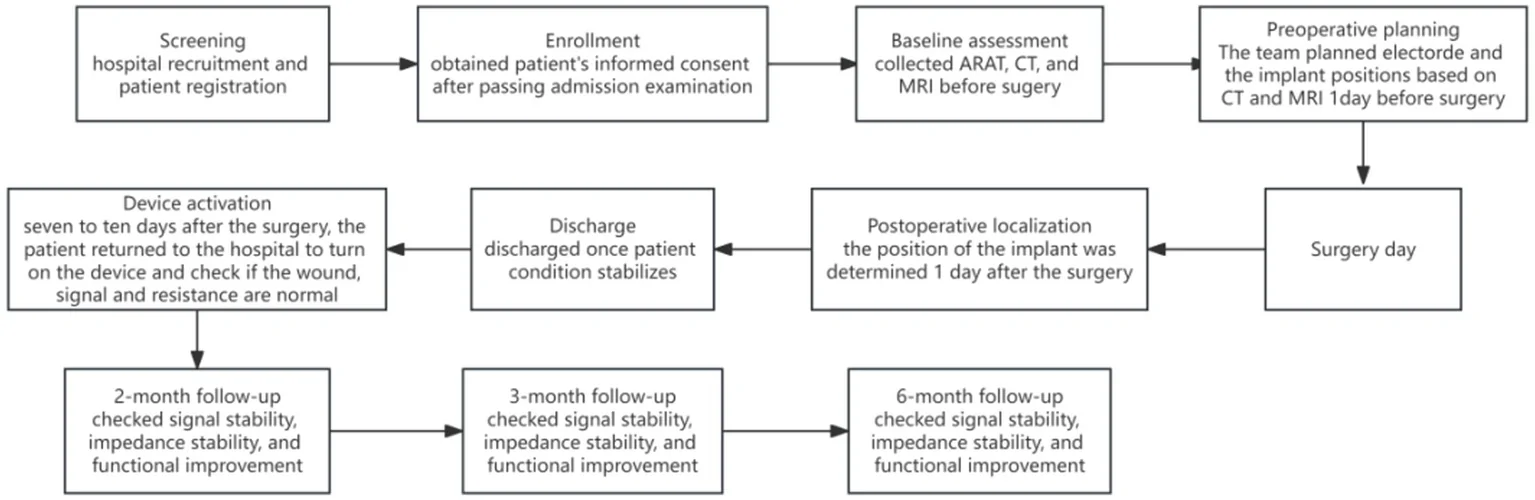
Flow chart of patient enrollment to postoperative follow-up.

Before surgery, the patient’s left-hand grasping score was 2 points. The device was activated on August 13, 2025, at which time all impedance values were within normal limits. The channel impedances were measured as 841, 976, 886, 732, 774, 926, 891, and 803*Ω*. At the 2-month follow-up on September 19, 2025, device impedance remained normal, with channel impedances of 1,327, 1,591, 1,556, 1,175, 1,516, 1,612, 1,489, and 1,489*Ω*. The patient’s left-hand BCIassisted grasping score was 17 points. At the 3-month follow-up, device impedance remained normal, with channel impedances of 1,482, 1,685, 1,612, 1,462, 1,647, 1,630, 1,604, and 1,552*Ω*. The patient’s left-hand BCI-assisted grasping score was 18 points. At the 6-month follow-up, device impedance remained normal, with channel impedances of 1,490, 1,646, 1,626, 1,489, 1,688, 1,698, 1,625, and 1,559*Ω*. The patient’s left-hand BCI-assisted grasping score was 18 points. No adverse events occurred following the brain–computer interface implantation, and the patient reported high satisfaction. The therapeutic flowchart is presented in Figure 2.

## 3 Perioperative management

### 3.1 Preoperative management

#### 3.1.1 Establishment of a multidisciplinary team and adequate preoperative preparation to ensure surgical quality

1. A multidisciplinary team was established, consisting of specialists from neurosurgery, psychology, anesthesiology, rehabilitation, and nursing.
2. Personnel training: Neurosurgeons and scrub nurses were required to possess sufficient experience in relevant implant procedures, complete product operation training preoperatively, and be fully familiar with all product labeling.
3. Psychological nursing: Preoperatively, the patient’s psychological status was evaluated using the Huaxi Emotional-index (HEI) developed by our hospital, administered by the “Sunshine Angels” (Wang et al., 2017). The HEI is designed for the rapid screening of emotional status including anxiety, depression, and suicide risk. The patient scored 11 points, indicating mild negative emotions. Psychological nursing was provided on the basis of routine care. Communication focused on topics of interest to the patient unrelated to his medical condition. The patient was encouraged to express his emotional and daily living status following spinal cord injury, thereby enabling a comprehensive understanding of his psychological state and identification of the underlying causes of negative emotions. Targeted comfort measures and psychological counseling were provided to enhance his willingness to accept treatment. Additionally, family members were encouraged to actively participate in the treatment process to address the patient’s needs for love and belonging.
4. Preoperative education: On the day prior to surgery, the operating surgeon explained the surgical approach and procedure to the patient, and the anesthesiologist instructed the patient on the requirements and purpose of preoperative fasting, thereby improving the patient’s understanding of the operation.
5. Preoperative localization: Based on the most recent cranial CT scan, the neurosurgeon selected attachment sites following the principles of “staying away from the surgical field, wide and even distribution, and bony stability.” In this case, 5–8 fiducial markers were attached over the frontal lobe, bilateral temporal bones, and parietal lobe. On the day before surgery, the patient underwent cranial CT scanning with the head positioned in the standard neutral orientation. Repeated scans and adjustments of marker positions were performed in the CT suite as needed (Ding and Wang, 2025). The patient was instructed on the following precautions: ① No touching or pressing: The patient was strictly prohibited from touching, pressing, scratching, or attempting to adjust the fiducial markers with hands or any objects. ② No hair washing or water exposure: Showering, hair washing, and any contact between the markers and water were absolutely forbidden. ③ Clothing and activity precautions: The patient was advised to dress and undress slowly and wear loose-necked clothing to avoid scratching the markers. Vigorous activities that may cause sweating were avoided to prevent the markers from detaching due to moisture. ④ Sleep position adjustment: The patient was instructed to maintain a supine position during sleep as much as possible. If lateral positioning was necessary, the side with fiducial markers had to be avoided to minimize friction and compression during sleep. ⑤ Monitoring and prompt reporting: The patient was instructed to immediately notify the attending physician or nurse upon observing any loosening, displacement, edge lifting, or detachment of the fiducial markers, and was strongly advised against reattaching the markers independently.
6. Preoperative skin preparation: On the day before surgery, the responsible nurse instructed the patient to undergo preoperative scalp disinfection. The scalp was first cleansed with regular shampoo to remove organic substances such as oil, dirt, and dandruff. A second scrub was then performed using a disinfectant containing 1.8–2.2% chlorhexidine gluconate for 5 min, followed by thorough rinsing with warm water and drying with a clean towel (World Health Organization, 2018).

#### 3.1.2 Preparation of equipment and consumables

1. Equipment and instrument preparation: An implantable neural acquisition and stimulation device, an implantable cortical electrode kit (including cortical electrodes, blank electrodes, electrode dilators, tunnellers, torque wrenches, cable clips, fixing screws, two-wing anchors for cable fixators, and electrode protective sleeves), a cerebral neural signal processor, and a disposable surgical tool kit (including stimulator templates, bone grinding detectors, and screwdrivers) developed by Borui Kang Medical Technology Co., Ltd. were prepared, along with a routine neurosurgical instrument kit (craniotomy instruments, craniotomy electric drill), a neuro-navigation system, and neuroelectrophysiological monitoring equipment.
2. Consumables preparation: Disposable consumables including gauze, gauze balls, irrigators, suction tubes, electrosurgical unit cleaning sheets, blades, cotton sheets (sizes: 0.8, 1.5, and 2.5 cm), gelatin sponges, and antibacterial films were prepared, as well as high-value consumables such as bipolar irrigating electrocoagulation forceps, push-button electrosurgical pencils, skull fixation materials, and absorbable sutures. Preoperatively, the integrity of surgical instruments and consumables, the absence of packaging damage, and the completeness of quantities were inspected. Normal saline at 37 °C (for intraoperative cortical irrigation) and distilled water (for testing contacts after electrode placement) were prepared in advance. Furthermore, the immediate availability of emergency equipment and drugs (e.g., antiepileptic drugs, vasopressors) was verified.

### 3.2 Intraoperative management

#### 3.2.1 Proper coordination with neuro-navigation for accurate electrode implantation

1. Acquisition of high-precision images: Preoperative high-resolution MRI was obtained for detailed visualization of brain tissue, lesions, and functional areas, and high-resolution CT was performed for clear depiction of cranial osseous anatomy.
2. Image fusion: Neurophysiology technicians downloaded MRI and CT images into the neuro-navigation system for precise registration and fusion, generating a three-dimensional dataset integrating both soft-tissue details and bony structures.
3. Navigation registration: After induction of general anesthesia, the patient’s head was fixed with head pins by the surgeon. The optical positioning camera of the neuro-navigation system was positioned 1–2 meters away from the surgical field. The reference frame was firmly mounted on the head clamp by neurophysiology technicians. A navigation probe equipped with reflective markers was calibrated by touching the reference frame. The technician then touched the center of each fiducial marker on the scalp sequentially; for each accurate contact, an assistant clicked the corresponding marker icon on the navigation interface. After the system received at least 4 pairs of “physical-virtual” matching points, it automatically aligned the acquired surface contour with the 3D model reconstructed from preoperative images, establishing the spatial correspondence between the virtual image space and the real surgical space.
4. Verification and confirmation: Multiple stable and unambiguous anatomical landmarks (e.g., nasion, medial and lateral canthi, tragus) were touched with the navigation probe to verify whether the virtual probe cursor on the navigation screen corresponded precisely to the anatomical locations on the images.
5. Real-time navigation: An opening was reserved in the long surgical draping to accommodate the navigation arm, which was then covered with a sterile drape to maintain the sterile field. The circulating nurse properly positioned the navigation equipment and secured the reflective markers on the probe. Navigation was performed 4 times to confirm the location of the precentral and postcentral gyri: before skin incision, after raising the scalp flap, before epidural electrode placement, and after electrode implantation, to ensure accurate electrode positioning.

#### 3.2.2 Strict prevention of incision infection and optimization of defense mechanisms

1. Immediate preoperative skin preparation: In the operating preparation area, the surgeon used a skin-friendly electric hair clipper to remove hair from the surgical field, a standard surgical procedure that achieves both infection prevention and humanistic care (World Health Organization, 2018).
2. Disinfection: After satisfactory general anesthesia, the skin was wiped with 75% ethanol to remove surface oils and contaminants. After complete drying, povidone-iodine was applied in concentric circles from the incision outward for 3 rounds. Following repositioning the bone flap, the bone flap and skin flap were irrigated with povidone-iodine. The incision was again wiped with povidone-iodine prior to scalp suturing (World Health Organization, 2018).
3. Application of disposable antibacterial surgical film: After the skin disinfectant had completely dried, a disposable antibacterial surgical film was applied to the surgical area. The film was spread and smoothed from the center of the incision outward to ensure tight adhesion to the skin without air bubbles. Surgical drapes were then fixed over it to form an active antibacterial sealing layer, which further enhanced sustained inhibition of residual skin flora and prevention of fluid penetration (Hu et al., 2015).
4. Antibiotic administration: Cefazolin sodium (1 g) was intravenously infused 0.5–1 h before surgery, with an additional dose administered 3 h after incision. Antibiotic administration was continued for 4–6 h postoperatively after the patient returned to the ward (World Health Organization, 2018).
5. Use of disposable waterproof surgical drapes: Intraoperative procedures, including drilling and irrigation, may wet sterile drapes and increase infection risk. Disposable waterproof surgical drapes are water-resistant and do not become porous after repeated use or reprocessing, thus effectively blocking microbial penetration.
6. Strict personnel and aseptic management: The circulating nurse supervised compliance with aseptic techniques among operating room staff and controlled the number of personnel in the operating room to reduce the risk of surgical site infection.

### 3.3 Postoperative management

#### 3.3.1 Standardized postoperative monitoring to ensure nursing quality and safety

1. Urinary and fecal care: The patient had sensory loss below the C3 level, with no awareness of the urge to urinate or defecate and an inability to control bowel and bladder functions, resulting in neurogenic bladder and neurogenic bowel. A planned and proactive management strategy was adopted. The nurse performed intermittent catheterization every 4–6 h to ensure that bladder capacity did not exceed 400–500 mL. Intermittent catheterization can reduce the risk of infection, protect bladder function and the upper urinary tract, and improve the patient’s quality of life and dignity (Wang et al., 2025). Concurrently, tolterodine tartrate tablets and mirabegron sustained-release tablets were administered to alleviate symptoms of overactive bladder (urgency and frequency). Bowel evacuation was performed every 1–2 days to stimulate defecation, generally 30–60 min after meals, and defecation was assisted by gravity while using a toilet or bedpan whenever possible.
2. Pain management: The patient developed pain at the cranial surgical site in the early morning after surgery, with a Visual Analog Scale (VAS) score of 6 points, indicating moderate pain. The pain was distending in nature, moderately affected daily life, and caused difficulty in falling asleep. As prescribed by the physician, 60 mg of loxoprofen sodium tablets were administered orally. The pain was relieved 1 h later, with a VAS score of 2 points, indicating mild pain. The patient rested quietly through the night and reported no further discomfort.
3. Rehabilitation training: ① Range of motion training: slow shoulder rotation and lateral flexion; active or passive flexion and extension of the shoulders, elbows, wrists, and fingers; and passive or assisted activities of the lower extremity joints (hips, knees, and ankles). ② Muscle strengthening training: upper extremity resistance training (using resistance bands, light dumbbells, sandbags, etc.); isometric contraction exercises of the neck muscles (pushing against walls, bed heads, etc.); and auxiliary muscle contraction using electrical stimulators. ③ Hand function training: passive or assisted finger stretching, and grip training using assistive devices (e.g., grip balls, resistance rings). ④ Activities of daily living training: practice of eating, washing, and other daily movements, as well as completing daily tasks with the help of assistive tools. ⑤ Transfer and position management: regular turning over, and training of sitting balance and trunk stability. ⑥ Respiratory training: deep breathing training (abdominal breathing) and use of respiratory trainers. ⑦ Assistive device use training: wheelchair operation and transfer training.
4. Orthostatic hypotension occurred as a result of impaired sympathetic nerve control following spinal cord injury, which compromised effective vascular contraction. Accordingly, the patient was assisted in sitting up slowly and progressively, and midodrine hydrochloride was administered as prescribed to elevate blood pressure.
5. Psychological care: Following brain–computer interface surgery, the patient had high expectations for future recovery; however, the rehabilitation process was lengthy. When treatment effects were not immediately apparent, the patient experienced negative emotions such as anxiety, depression, and discouragement. Nurses set short-term, achievable goals for the patient and acknowledged even minor improvements with affirmation and congratulations. Successful cases were introduced to boost the patient’s self-confidence. The patient was encouraged to freely express feelings, difficulties, and fears, while the nurse listened attentively and provided positive responses.
6. Prevention of complications: ① Pressure injury: The patient was at moderate risk for pressure injury. The responsible nurse assisted the patient with regular repositioning, applied pressure-relieving patches to bony prominences, and used an alternating pressure air mattress. ② Lower extremity venous thrombosis: The patient was encouraged to engage in active or passive training. ③ Incontinence-associated dermatitis: Immediately after each incontinence episode, the skin was gently cleaned with a pH-balanced cleanser using a patting motion (avoiding friction) and thoroughly dried by patting. Subsequently, a barrier cream containing zinc oxide was routinely applied to the skin, or a liquid dressing was sprayed to form a protective barrier. Highly absorbent pads with superior fluid retention were selected and replaced in a timely manner to ensure that the skin remained clean, dry, and protected.

#### 3.3.2 Conducting discharge follow-up to improve the quality of continuous nursing

1. Discharge instructions: ① Avoid respiratory infections and exposure to cold, intensify rehabilitation training, and optimize nutritional intake as appropriate. ② Change the cranial wound dressing every 2–3 days, and remove sutures 2 weeks postoperatively depending on the condition of wound healing. ③ Perform the first postoperative cranial CT re-examination at 3 months after surgery, followed by re-examinations every 3–6 months according to clinical necessity.
2. Telefollow-up: A telefollow-up channel was established using the WeChat platform, with follow-up conducted every 2 weeks. The follow-up content included the following: ① Device-related follow-up: Inquiry was made regarding whether the patient experienced any foreign body sensation, pain, or discomfort. ② Physiological and safety follow-up: Signs of infection, such as redness, swelling, warmth, pain, or exudation at the scalp incision, were closely monitored. The skin in contact with the electrodes was checked for allergies, pressure ulcers, or inflammation. Possible implant-related serious adverse events, including seizures, headaches, and newly developed neurological deficits, were assessed and inquired about. ③ Function and rehabilitation follow-up: A personalized home-based rehabilitation training plan was formulated according to the patient’s condition. The patient was encouraged to actively participate in rehabilitation exercises to stimulate motor nerve recovery and remodeling, ensuring the intensity, effectiveness, and accuracy of the training. ④ Psychological follow-up: The patient’s emotional state was regularly assessed, including whether feelings of depression, anxiety, disappointment, or “technological dependence” occurred when performance was suboptimal. Psychological counseling was provided, expectations of the patient and family members were managed, and they were encouraged to maintain patience and a positive attitude. Every small improvement was celebrated to enhance the patient’s self-efficacy.
3. Emergency support: A dedicated hotline contact was clearly provided to ensure that the patient could obtain immediate guidance in the event of an emergency situation (e.g., severe discomfort, device malfunction).

## 4 Conclusion

Patients with TSCI constitute a population that merits extensive societal attention and concern. These individuals experience a sudden transition from an able-bodied state to disability, confronting multifaceted and complex challenges spanning physiological, psychological, social, and economic domains. To systematically address these challenges, the core strategy lies in the provision of high-quality, continuous medical care and nursing intervention. Preoperatively, a multidisciplinary collaborative team should be established to facilitate thorough preparation and ensure optimal surgical quality. Intraoperatively, the scrub nurse should actively support neuro-navigation to guarantee precise electrode implantation, rigorously prevent the risk of incisional infection, and reinforce aseptic defensive mechanisms. Postoperatively, enhanced monitoring should be implemented to safeguard nursing quality and safety, and follow-up after discharge should be conducted to improve the quality of transitional care.

This case represents the first surgical navigation-guided BCI implantation performed in western China. No adverse events were observed during the 6-month postoperative follow-up, providing preliminary clinical evidence supporting the short-term safety of this surgical approach. However, given the inherent limitations of a single-case report, the generalizability of the perioperative management strategy to SCI patients with varying injury characteristics warrants cautious interpretation. Furthermore, critical issues pertaining to long-term electrode functional stability and host tissue biocompatibility necessitate extended follow-up beyond the current observation window. Future research should prioritize prospective cohort studies and multicenter collaborative efforts to formulate standardized, evidence-based guidelines, thereby facilitating the transition of this technique from isolated case exploration to broader clinical implementation.

## Data Availability

The original contributions presented in the study are included in the article/Supplementary material, further inquiries can be directed to the corresponding author.
