# Correction: Perioperative management for invasive brain–computer interface implantation under surgical navigation in a patient with traumatic spinal cord injury: a case report

**DOI:** 10.3389/fnhum.2026.1995038

**Published:** 2026-10-09

**Authors:** Shuhui Yang, Qiang Sun, Hong Mo, Wenyao Cui

**Affiliations:** 1 Department of Neurosurgery, West China Hospital, Sichuan University, Chengdu, China; 2 West China School of Nursing, Sichuan University, Chengdu, China; 3 Department of Anesthesiology, West China Hospital, Sichuan University, Chengdu, China

**Keywords:** brain–computer interface, nursing cooperation, perioperative management, spinal cord injury, surgical navigation

In the original publication, the ARAT scores were reported without clearly specifying the assessment condition (with or without BCI assistance). Upon post-publication review, the authors identified that the reported scores should have been described as those obtained with the assistance of the BCI system. The text has been revised accordingly to accurately reflect the assessment condition and to report the correct left-hand grasp ARAT score with BCI assistance.

A correction has been made to the section (1) the **Case summary** in the **Abstract**; (2) Point 2 in the section “**1 What does this paper contribute to the wider global clinical community?**”; and (3) the second-to-last paragraph of subsection “**2.2 Treatment course and outcomes**” under section 2 Clinical materials:

“(1) At the 6-month follow-up, the patient's left-hand brain–computer interface-assisted grasping score was 18 points.; (2) At the 6-month follow-up, the patient's left-hand brain–computer interface-assisted grasping score was 18 points. This finding is expected to promote the wide application of brain–computer interfaces in patients with spinal cord injuries; (3) Before surgery, the patient's left-hand grasping score was 2 point. The device was activated on August 13, 2025, at which time all impedance values were within normal limits. The channel impedances were measured as 841, 976, 886, 732, 774, 926, 891, and 803 Ω. At the 2-month follow-up on September 19, 2025, device impedance remained normal, with channel impedances of 1,327, 1,591, 1,556, 1,175, 1,516, 1,612, 1,489, and 1,489 Ω. The patient's left-hand BCI-assisted grasping score was 17 points. At the 3-month follow-up, device impedance remained normal, with channel impedances of 1,482, 1,685, 1,612, 1,462, 1,647, 1,630, 1,604, and 1,552 Ω. The patient's left-hand BCI-assisted grasping score was 18 points. At the 6-month follow-up, device impedance remained normal, with channel impedances of 1,490, 1,646, 1,626, 1,489, 1,688, 1,698, 1,625, and 1,559 Ω. The patient's left-hand BCI-assisted grasping score was 18 points. No adverse events occurred following the brain–computer interface implantation, and the patient reported high satisfaction. The therapeutic flowchart is presented in **Figure 2**.”

The original version of this article has been updated.

