# Supplementary material for: Perioperative management for invasive brain–computer interface implantation under surgical navigation in a patient with traumatic spinal cord injury: a case report
Source: Front Hum Neurosci. 2026 Aug 25;20:1892331. doi: 10.3389/fnhum.2026.1892331 (PMC13550118; doi:10.3389/fnhum.2026.1892331)
Supplement: Supplementary file 1 [file Data_Sheet_1.pdf]

CARE Checklist – 2016: Information for writing a case report

Topic	Item	Checklist item description	Line/Page
**Title**	1	The words "case report" should be in the title along with the area of focus	title
**Key Words**	2	Four to seven key words—include "case report" as one of the key words	keywords
**Abstract**	3a	Background: What does this case report add to the medical literature?	abstract
	3b	Case summary: chief complaint, diagnoses, interventions, and outcomes	abstract
	3c	Conclusion: What is the main "take-away" lesson from this case?	abstract
**Introduction**	4	The current standard of care and contributions of this case—with references (1-2 paragraphs)	introduction
**Timeline**	5	Information from this case report organized into a timeline (table or figure)	figure2,
**Patient Information**	6a	De-identified demographic and other patient or client specific information	general information
	6b	Chief complaint—what prompted this visit?	general information
	6c	Relevant history including past interventions and outcomes	general information
**Physical Exam**	7	Relevant physical examination findings	general information
**Diagnostic Assessment**	8a	Evaluations such as surveys, laboratory testing, imaging, etc.	treatment course and outcomes
	8b	Diagnostic reasoning including other diagnoses considered and challenges	treatment course and outcomes
	8c	Consider tables or figures linking assessment, diagnoses and interventions	treatment course and outcomes
	8d	Prognostic characteristics where applicable	no applicable
**Interventions**	9a	Types such as life-style recommendations, treatments, medications, surgery	nursing care
	9b	Intervention administration such as dosage, frequency and duration	nursing care
	9c	Note changes in intervention with explanation	no change
	9d	Other concurrent interventions	nursing care
**Follow-up and Outcomes**	10a	Clinician assessment (and patient or client assessed outcomes when appropriate)	treatment course and outcomes
	10b	Important follow-up diagnostic evaluations	treatment course and outcomes
	10c	Assessment of intervention adherence and tolerability, including adverse events	treatment course and outcomes
**Discussion**	11a	Strengths and limitations in your approach to this case	conclusion
	11b	Specify how this case report informs practice or Clinical Practice Guidelines (CPG)	conclusion
	11c	How does this case report suggest a testable hypothesis?	conclusion
	11d	Conclusions and rationale	conclusion
**Patient Perspective**	12	When appropriate include the assessment of the patient or client on this episode of care	treatment course and outcomes
**Informed Consent**	13	Informed consent from the person who is the subject of this case report is required by most journals	The patient's informed consent has been obtained.
**Additional Information**	14	Acknowledgement section; Competing Interests; IRB approval when required	title page
